# Supplementary material for: Initial results with an absorbable urologic scaffold to mitigate early urinary incontinence following radical prostatectomy: the ARID study
Source: World J Urol. 2026 Jan 17;44(1):106. doi: 10.1007/s00345-026-06186-7 (PMC12812077; doi:10.1007/s00345-026-06186-7)
Supplement: Supplementary file 1 — Supplementary Material 1 [file 345_2026_6186_MOESM1_ESM.pdf]

Supplementary Material

Initial Results with an Absorbable Urologic Scaffold to Mitigate Early Urinary Incontinence Following Radical Prostatectomy: The ARID Study

Supplementary Table 1. Summary of Change in 24-Hour Pad Weights Over Time

Subject Number	Device Placement	Pre-procedure Pad Weight (grams)	Difference in 24 Hour Pad Weight Measurement Post-Radical Prostatectomy vs. Baseline (grams)			
			Removal	4-6 Weeks	3 Months	6 Months
1	Full Extension	15.2	*	2.8	-1.5	31.4
2	Limited Extension	10.5	*	308.3	78.2	19.2
3	Full Extension	37.1	*	-1.7	-15.5	-7.7
4	Full Extension	25.7	*	16.4	96.7	-3.3
5	Full Extension	19.6	*	34.6	2.0	6.9
6	Full Extension	18.0	0.2	3.1	-3.0	-6.1
7	Limited Extension	37.8	434.0	815.1	619.8	419.7
8	Full Extension	23.0	3.7	3.6	0.6	0.4
9	Full Extension	37.1	-27.6	-12.4	-11.8	-16.4
10	Limited Extension	29.3	445.1	757.6	497.7	8.7
11	Full Extension	34.2	25.0	40.3	3.8	0.2
12	Full Extension	26.8	6.4	2.0	-3.2	-5.3
13	Full Extension	32.2	8.6	-13.7	2.0	4.2
14	Limited Extension	8.0	685.6	391.4	679.3	405.9
15	Full Extension	32.8	179.8	-4.0	64.2	91.4
16	Limited Extension	9.2	232.9	428.5	285.4	195.2
17	Full Extension	28.0	-1.3	-2.8	0.4	2.9
18	Full Extension	22.1	17.5	62.8**	27.0	31.3
19	Limited Extension	15.5	206.0	90.6	42.6	57.6
20	Full Extension	21.1	-6.3	-0.3	-2.5	-8.3
21	Limited Extension	20.1	191.0	173.0	52.7	16.9
22	Full Extension	45.8	58.9	-37.4	***	***
23	Full Extension	45.2	105.4	208.0	***	***
24	Limited Extension	2.6	320.9	319.3	***	***

* Visit not required for subjects initially enrolled in a previous version of the study protocol

** Not included in the analysis because subject presented with a meatal stenosis at the penis tip due to catheter placement

*** Data not yet available at time of cutoff date for present report

Supplementary Table 2. Summary of Change in 1-Hour Pad Weights Over Time

Subject Number	Device Placement	Pre-procedure Pad Weight (grams)	Difference in 24 Hour Pad Weight Measurement Post-Radical Prostatectomy vs. Baseline (grams)		
			4-6 Weeks	3 Months	6 Months
1	Full Extension	0	0.7	0	0
2	Limited Extension	0	41	88.7	0
3	Full Extension	1.7	1.6	-1	9.2
4	Full Extension	1	34.8	73	0.7
5	Full Extension	0	0.2	0	0
6	Full Extension	0	2.6	10.5	0
7	Limited Extension	0.4	75.5	57.7	24.9
8	Full Extension	0	0.7	0.2	0
9	Full Extension	0	0.6	1.4	1
10	Limited Extension	0	66.1	174.1	42.2
11	Full Extension	0	2.3	0.3	2.3
12	Full Extension	0	0.3	0	0
13	Full Extension	0	0.7	4	0
14	Limited Extension	0.1	63.4	100.8	157.5
15	Full Extension	0	1.6	8.4	9.8
16	Limited Extension	0	53	18.2	32
17	Full Extension	16.7	-15.8	-16	-12.9
18	Full Extension	0	102.7*	2.4	20
19	Limited Extension	0	21.6	128	72.2
20	Full Extension	0	1.7	0	1.5
21	Limited Extension	0	22.5	0.4	7.2
22	Full Extension	0	0	**	**
23	Full Extension	0	4.8	**	**
24	Limited Extension	0.3	18.1	**	**

* Not included in the analysis because subject presented with a meatal stenosis at the penis tip due to catheter placement
** Data not yet available at time of cutoff date for present report
